# Multivariate Modeling Identifies Neutrophil- and Th17-Related Factors as Differential Serum Biomarkers of Chronic Murine Colitis

**DOI:** 10.1371/journal.pone.0013277

**Published:** 2010-10-19

**Authors:** Megan E. McBee, Yu Zeng, Nicola Parry, Cathryn R. Nagler, Steven R. Tannenbaum, David B. Schauer

**Affiliations:** 1 Biological Engineering Department, Massachusetts Institute of Technology, Cambridge, Massachusetts, United States of America; 2 Division of Comparative Medicine, Massachusetts Institute of Technology, Cambridge, Massachusetts, United States of America; 3 Department of Pathology, The University of Chicago, Chicago, Illinois, United States of America; University of California Los Angeles, United States of America

## Abstract

**Background:**

Diagnosis of chronic intestinal inflammation, which characterizes inflammatory bowel disease (IBD), along with prediction of disease state is hindered by the availability of predictive serum biomarker. Serum biomarkers predictive of disease state will improve trials for therapeutic intervention, and disease monitoring, particularly in genetically susceptible individuals. Chronic inflammation during IBD is considered distinct from infectious intestinal inflammation thereby requiring biomarkers to provide differential diagnosis. To address whether differential serum biomarkers could be identified in murine models of colitis, immunological profiles from both chronic spontaneous and acute infectious colitis were compared and predictive serum biomarkers identified via multivariate modeling.

**Methodology/Principal Findings:**

Discriminatory multivariate modeling of 23 cytokines plus chlorotyrosine and nitrotyrosine (protein adducts from reactive nitrogen species and hypochlorite) in serum and tissue from two murine models of colitis was performed to identify disease-associated biomarkers. Acute *C. rodentium*-induced colitis in C57BL/6J mice and chronic spontaneous *Helicobacter-*dependent colitis in TLR4^−/−^ x IL-10^−/−^ mice were utilized for evaluation. Colon profiles of both colitis models were nearly identical with chemokines, neutrophil- and Th17-related factors highly associated with intestinal disease. In acute colitis, discriminatory disease-associated serum factors were not those identified in the colon. In contrast, the discriminatory predictive serum factors for chronic colitis were neutrophil- and Th17-related factors (KC, IL-12/23p40, IL-17, G-CSF, and chlorotyrosine) that were also elevated in colon tissue. Chronic colitis serum biomarkers were specific to chronic colitis as they were not discriminatory for acute colitis.

**Conclusions/Significance:**

Immunological profiling revealed strikingly similar colon profiles, yet distinctly different serum profiles for acute and chronic colitis. Neutrophil- and Th17-related factors were identified as predictive serum biomarkers of chronic colitis, but not acute colitis, despite their presence in colitic tissue of both diseases thereby demonstrating the utility of mathematical modeling for identifying disease-associated serum biomarkers.

## Introduction

Intestinal inflammation develops from known causes such as infection with enteropathogenic *E. coli* (EPEC) or from unknown causes as in inflammatory bowel diseases (IBD). Compared to the chronic idiopathic intestinal inflammation that occurs in IBD patients, intestinal infections cause acute colitis that is resolved by host defenses. A need for biomarkers that predict the presence and severity of intestinal disease remains despite the individual association of several non-disease related proteins (such as C-reactive protein or antibodies against *E. coli* OmpC and glycans) with chronic intestinal disease [1]–[6]. Identification of disease-relevant serum biomarkers discriminating chronic colitis from other conditions, such as acute infectious colitis, or biomarkers identifying relative disease severity allowing non-invasive monitoring of disease progression and responsiveness to therapeutic treatments remain elusive.

To examine immunological factors associated with both acute and chronic intestinal disease, two murine models, one of acute infectious colitis and the other of chronic spontaneous colitis, were studied. *Citrobacter rodentium*, a murine pathogen that recapitulates much of the pathology seen in human EPEC infection, causes acute infectious colitis. *C. rodentium*-induced colitis is characterized by epithelial hyperplasia, erosion and destruction of the epithelial brush border, edema, and inflammation [7]. In C57BL/6 mice, *C. rodentium* infection is self-resolving with pathology peaking at 2 weeks post-infection (WPI) and disease resolution by 4-6 WPI [8]. Immune mediators in *C. rodentium-*induced colitis have been extensively studied in mice with targeted knockouts of innate and adaptive cells, as well as cytokines, cytokine receptors, and pattern recognition receptors. These studies have shown that bacterial clearance and disease resolution require both protective antibodies and an IFN-γ mediated T effector cell response [9]–[12] whereas other immunological mediators prevent early mortality through maintenance of epithelial barrier function [13]–[16]. Th17 cells are generated in abundance during infection, with IL-17 production peaking with maximal disease; however IL-17 is not required for survival or bacterial clearance and its role in disease pathogenesis is still not well understood [8], [13], [17]. The common finding of these studies has been the identification of a robust inflammatory response in the colon characterized by the increased production of the inflammatory mediators TNF-α, IFN-γ, IL-1β, IL-17, and IL-6.

In contrast to acute *C. rodentium*-induced colitis, chronic spontaneous typhlocolitis develops in IL-10^−/−^ mice colonized with *Helicobacter spp.* [18]. *Helicobacter spp.*-positive (Hsp^+^) IL-10^−/−^ mice also deficient in TLR4 (TLR4^−/−^ x IL-10^−/−^ [DKO]) exhibit earlier onset and increased severity of typhlocolitis, which is dependent upon infection with *Helicobacter spp.* [19]. Interestingly, inflammatory mediators in colonic cultures from colitic DKO mice were similar to those found in colon tissue during acute *C. rodentium-*induced colitis: TNF-α, IFN-γ, IL-1β, IL-17, and IL-6 [19]. Therefore, a comprehensive study comparing the direct ex-vivo colonic cytokine protein profile with matched serum cytokine profile from the two forms of murine colitis was conducted in order to identify predictors of intestinal disease severity, specifically potential serum biomarkers of chronic intestinal inflammation. The protein adducts nitrotyrosine (NT) and chlorotyrosine (CT) along with 23 cytokines, measured in serum and colon of wild type C57BL/6J mice with acute infectious *C. rodentium*-induced colitis and DKO mice with chronic spontaneous *Helicobacter*-dependent typhlocolitis, were evaluated for their discriminatory power and ability to predict intestinal disease severity, and thereby their potential usefulness as biomarkers.

## Results

### Robust, comparable colitis in acute infectious and chronic spontaneous models

**Figure 1:**
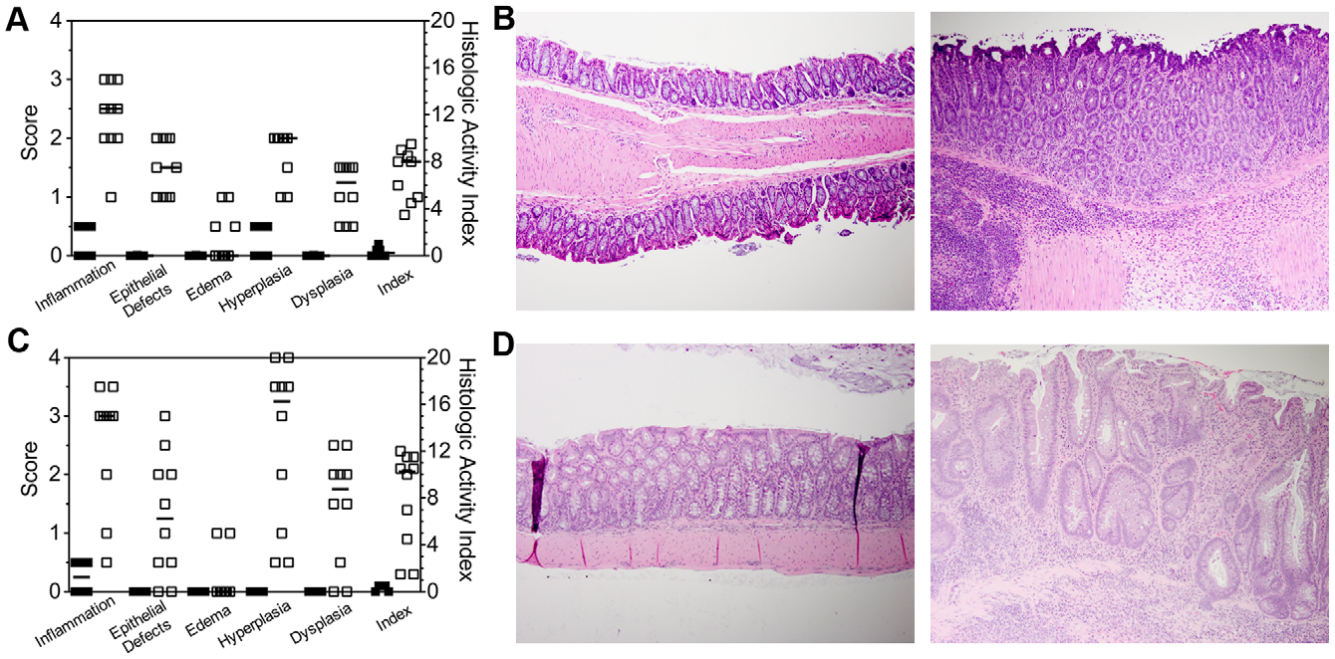
Marked colitis in *C. rodentium* infected and aged TLR4^−/−^ x IL-10^−/−^ (DKO) mice colonized with *Helicobacter* spp. Histological scores for inflammation, epithelial defects, edema, hyperplasia, and dysplasia in the colon for uninfected (Cr^−^, ▪) and *C. rodentium* infected (Cr^+^, □) mice at 14 DPI (A) and *Helicobacter spp*. negative (Hsp^−^, ▪) or positive (Hsp^+^, □) aged DKO mice (C). All scores were summed to form the histological activity index for individual mice. All pairings are *** *P*<0.0001 by Mann-Whitney test. (B) Representative H&E stained colon section from Cr^−^ (left panel) and Cr^+^ (right panel) mice at 14 DPI. (D) Representative H&E stained colon section from Hsp^−^ (left panel) and Hsp^+^ (right panel) DKO mice. Original magnification 100×.

Infection with *C. rodentium* was monitored for 14 days post-infection (DPI) with peak bacterial burdens of 9×10^8^ CFU/g feces at 4 DPI, **Figure S1A**. Development of disease was monitored by change in body weight with *C. rodentium* infected (Cr^+^) mice losing 3% of initial body weight by 14 DPI compared with uninfected mice gaining 4% (*P*<0.01, **Figure S1B)**. At 14 DPI histological findings included increased inflammatory infiltrates, epithelial defects, edema, hyperplasia, and dysplasia (**Figure 1A and 1B**). These five categorical lesions were scored and summed to form the histologic activity index (HAI). Marked colitis was present in Cr^+^ mice (**Figure 1A and 1B**) with a median HAI of 8.0 (range 3.5 to 9.5), compared with 0.25 (0–1.0) in uninfected mice.

The age of onset of chronic spontaneous colitis in Hsp^+^ DKO mice is variable, therefore colitis was evaluated when >30% of mice have rectal prolapse [19]. Gross evaluation revealed no disease in Hsp^−^ DKO mice, whereas Hsp^+^ DKO mice had poor body condition with colonic and cecal thickening. Histological findings were similar to acute colitis (**Figure 1C and 1D**) plus focal gland herniation into the muscularis mucosa in 3 of 10 mice. Hsp^−^ mice had a median HAI of 0.5 (range 0–0.5) while Hsp^+^ mice had a median HAI of 10.25 (range 1.5–12), **Figure 1C**.

### Local and systemic cytokine profiles in acute colitis indicate robust inflammation

**Figure 2:**
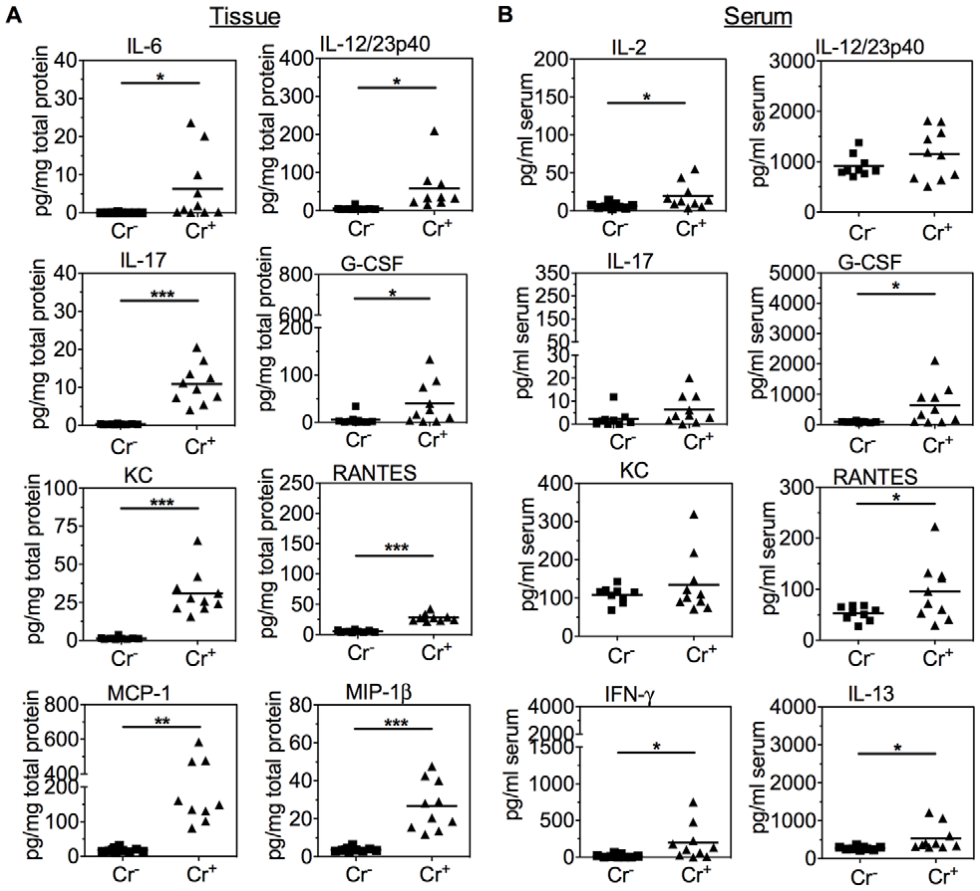
Colonic and serum cytokines associated with acute *C. rodentium*-induced colitis. Cytokines in colon tissue (A) and serum (B) of uninfected (Cr^−^; n = 10 tissue, n = 9 serum) and *C. rodentium* infected (Cr^+^; n = 10) mice at 14 DPI. Colon values were normalized to total protein. Line indicates mean value. * *P*<0.05, ** *P*<0.01, *** *P*<0.001 by unpaired Student's t-test.

The complex colonic cytokine milieu present during peak severity of acute *C. rodentium* colitis has not previously been analyzed in detail at the protein level. To gain additional biological insight into the active disease process 23 cytokines from frozen full-thickness colon sections at 14 DPI were analyzed. Chemokines KC and MCP-1 and the cytokines IL-1β, IL-6, IL-12/23p40, and IL-17 were elevated in colon tissue of Cr^+^ mice, **Figure 2A**, confirming previous studies performed at the mRNA level [8], [13], [14], [20]. Newly identified factors induced by *C. rodentium* infection are cytokines associated with T cell and neutrophil proliferation (IL-2 and G-CSF) and chemokines (RANTES, MIP-1α, and MIP-1β), **Figure 2A** **and Figure S2A**. Of the 23 cytokines measured only five were significantly elevated in the serum at 14 DPI, **Figure 2B** **and Figure S2B**. Of note was the elevation of IFN-γ in serum indicating, perhaps, a broader systemic role for this cytokine in disease resolution. Chemotactic and proliferation promoting cytokines G-CSF, IL-2, and RANTES were elevated in serum in addition to tissue, **Figure 2B**, indicating that the presence of acute intestinal inflammation is detectable both locally and systemically.

### Tissue cytokines in chronic typhlocolitis mimic acute colitis and are represented in serum

**Figure 3:**
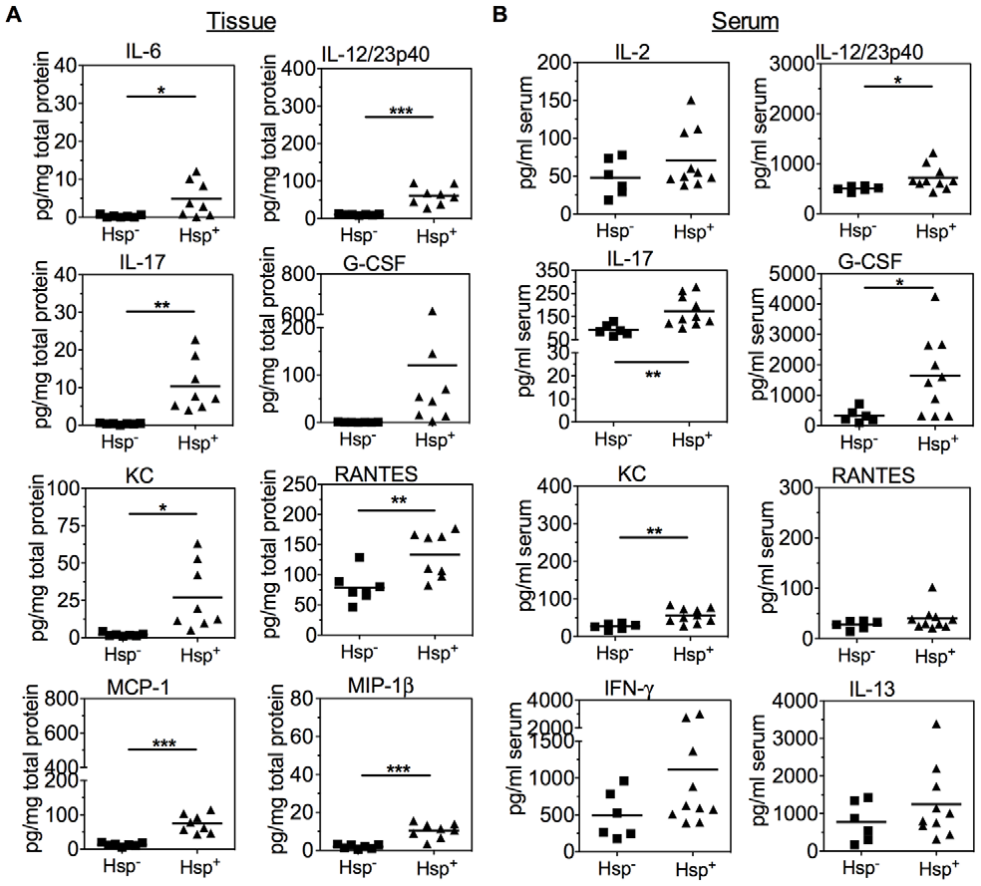
Colonic and serum cytokines associated with chronic *Helicobacter*-dependent colitis. Cytokines in colon tissue (A) and serum (B) in *Helicobacter*-free (Hsp^−^; n = 6) and *Helicobacter spp.*-positive (Hsp^+^; n = 8 tissue, n = 10 serum) TLR4^−/−^ x IL-10^−/−^ mice. Colon values were normalized to total protein. Line indicates mean value. * *P*<0.05, ** *P*<0.01, *** *P*<0.001 by unpaired Student's t-test.

Unlike *C. rodentium* colitis where inflammation develops within 2 weeks, chronic *Helicobacter*-dependent typhlocolitis develops over several months without resolving. The gradual recruitment and activation of immune cells to the intestines in chronic colitis, as well as the lack of TLR4 and IL-10 signaling, suggests activation of immune pathways and secretion of cytokines that might differ from acute colitis. Surprisingly, the cytokines elevated in colon tissue of Hsp^+^ DKO mice with chronic colitis compared with Hsp^−^ DKO mice mirrored those in acute colitis, **Figure 3A** **and Figure S3A**. This finding suggests that in chronic colitis not only is there sustained activation of acute inflammatory pathways, but also the continual presence and recruitment to the mucosa of the same cell types observed in acute colitis. In contrast to acute colitis, serum cytokines elevated in mice with chronic colitis were representative of those elevated in tissue: IL-6, IL-12/23p40, IL-17, G-CSF, and KC, **Figure 3B**. The combined elevation of these neutrophil- and Th17-associated factors in serum and tissue imply functional roles for these cells in chronic intestinal disease.

### Elevation of protein adducts from reactive nitrogen species and hypochlorite in colitic mice

**Figure 4:**
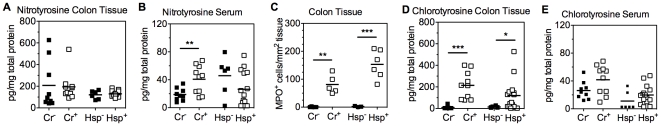
MPO-expressing cells in colon and level of protein adducts chlorotyrosine and nitrotyrosine in colon tissue and serum of both *C. rodentium* infected mice and Hsp^+^ TLR4^−/−^ x IL-10^−/−^ (DKO) mice. Nitrotyrosine levels in colon tissue (A) and serum (B) of uninfected (Cr^−^; n = 10 for tissue, n = 9 for serum) C57BL/6J mice, *C. rodentium* infected (Cr^+^; n = 10) C57BL/6J mice at 14 DPI, *Helicobacter*-negative (Hsp^−^; n = 6) DKO mice and *Helicobacter*-positive (Hsp^+^; n = 15) DKO mice at 20–35 weeks of age. (C) MPO^+^ cells in colon tissue of Cr^−^, Cr^+^, Hsp^−^, and Hsp^+^ (n = 5 per group) were counted in ten high power (40×) fields of colon tissue per mouse and averaged. Chlorotyrosine levels in colon tissue (D) and serum (E) from Cr^−^ (n  = 10 for tissue, n = 9 for serum), Cr^+^ (n = 10), Hsp^−^ DKO (n = 6) and Hsp^+^ DKO (n = 15). Adduct concentrations (A, B, D, E) were normalized to total protein in each sample. Line indicates mean value. * *P*<0.05, ** *P*<0.01, *** *P*<0.001 by unpaired Student's t-test.

Upon microbial activation, numerous reactive chemical species are produced by innate immune cells (including macrophages, neutrophils, and epithelial cells). Nitric oxide (NO) reacts with superoxide to form peroxynitrite, which can then react with tyrosine to form nitrotyrosine adducts (NT) [21]. Epithelial cells and colonic macrophages increase their production of NO during both acute *C. rodentium-*induced colitis and chronic *Helicobacter*-dependent colitis, although the relative contribution of NO from each cell type is unclear [22]–[25]. Therefore, NT was measured in colonic lysates as a marker for both macrophage infiltration and epithelial activation. NT levels in colons of Cr^+^ mice were comparable to uninfected mice, **Figure 4A**. Despite the robust chronic inflammation in Hsp^+^ DKO mice, there was no difference in NT levels compared with Hsp^−^ DKO mice, **Figure 4A**. NT was also measured in serum as a biomarker for peripheral tissue inflammation. In contrast to tissue, serum NT levels were significantly elevated in Cr^+^ mice when compared to the low levels detected in uninfected mice, **Figure 4B**. However, chronically inflamed DKO mice had no significant difference in serum NT, **Figure 4B**. Despite the presence of increased iNOS and NO during disease, protein adducts of other reactive nitrogen and oxygen species may provide better markers for chronic colitis.

Myeloperoxidase (MPO), the predominant protein in neutrophils involved with reactive oxygen species, generates hypochlorous acid from chloride ions and hydrogen peroxide [26]. Hypochlorous acid, in addition to killing bacteria, reacts with tyrosyl protein residues to form the stable adduct 3-chlorotyrosine (CT) [27]. Neutrophils are required for recovery from *C. rodentium*-induced colitis with both recruitment and MPO production peaking at 2 WPI [20], [28]. In the present study, MPO^+^ cells were not present in colons of either uninfected C57BL/6J or Hsp^−^ DKO mice **Figure 4C**, whereas colon tissue from both Cr^+^ C57BL/6J and Hsp^+^ DKO mice had significant increases in MPO^+^ cells, **Figure 4C**. As a biomarker of neutrophil presence and activity CT was measured in colon and serum. In agreement with the MPO staining, CT levels in colon from both uninfected C57BL/6J and Hsp^−^ DKO mice were low, **Figure 4D**, indicating minimal presence of neutrophils in non-colitic mice. A pronounced increase in CT was found in colons of both colitic Cr^+^ mice and Hsp^+^ DKO mice, **Figure 4D**. Serum CT was elevated in mice with either acute or chronic colitis compared with non-colitic mice, **Figure 4E**, but these increases did not reach statistical significance.

### Tissue and serum factors discriminate colitic from non-colitic mice

PLS-DA (Partial Least Squares Projection to Latent Structures-Discriminant Analysis) was used to determine the variables with the highest discriminatory power for colitic and non-colitic mice. For each type of colitis (acute and chronic) two separate PLS-DA models were generated: one with factors collected non-invasively (*serum*) using serum cytokine, CT, and NT levels and a second model (*tissue*) of invasively collected factors using colon tissue cytokine, CT, and NT levels. The acute *serum* model did not discriminate colitic from non-colitic mice with only 45.7% of the class distinction explained by the model's components, and only 32.9% of the variance among samples explained by the model, **Table 1**. The most influential factors or variables of importance (VIP) in the model included: NT, CT, recruitment and proliferation cytokines IL-2, G-CSF and RANTES, and a mix of T cell cytokines (IL-4, IL-17, IL-13, and IFN-γ), **Table 2**. The chronic *serum* model was able to discriminate colitic Hsp^+^ DKO mice from Hsp^−^ DKO mice with 84.0% of the class distinction and 69.1% of the variance explained by the model, **Table 1**. Most influential factors in the chronic *serum* model were significantly elevated and associated with both neutrophils and Th17 cells (KC, IL-17, IL-12/23p40, and G-CSF), **Table 2**.

**Table 1:** PLS-DA component contributions to discrimination (R^2^ Y) and variance (Q^2^) of colitic and non-colitic mice.

Acute Serum					Chronic Serum				
Component	R^2^ Y	R^2^ Y (cumulative)	Q^2^	Q^2^ (cumulative)	Component	R^2^ Y	R^2^ Y (cumulative)	Q^2^	Q^2^ (cumulative)
1	0.457	**0.457**	0.329	**0.329**	1	0.415	0.415	0.246	0.246
					2	0.425	**0.840**	0.590	**0.691**

Acute Colon					Chronic Colon				
Component	R^2^ Y	R^2^ Y (cumulative)	Q^2^	Q^2^ (cumulative)	Component	R^2^ Y	R^2^ Y (cumulative)	Q^2^	Q^2^ (cumulative)
1	0.883	0.883	0.829	0.829	1	0.832	0.832	0.798	0.798
2	0.056	0.939	0.368	0.892	2	0.073	0.905	0.207	0.840
3	0.052	**0.991**	0.524	**0.948**	3	0.079	**0.984**	0.344	**0.895**

**Table 2:** Most influential variables in acute and chronic colitis serum and colon tissue PLS-DA models by Variable of Importance in Projection (VIP) values.

**Acute Serum**														
**Variable**	G-CSF	NT	IL-2	IFN-γ	IL-4	IL-13	RANTES	IL-17	CT					
**VIP**	1.82	1.64	1.59	1.5	1.41	1.33	1.29	1.24	1.17					
**Acute Colon**														
**Variable**	KC	RANTES	IL-12/23p40	IL-1β	MIP-1β	MCP-1	IL-17	MIP-1α	IL-6	CT	IL-4			
**VIP**	1.48	1.44	1.43	1.40	1.39	1.28	1.26	1.23	1.18	1.18	1.14			
**Chronic Serum**														
**Variable**	Eotaxin	IL-12/23p40	KC	IL-17	G-CSF	IL-6								
**VIP**	1.51	1.45	1.42	1.17	1.16	1.03								
**Chronic Colon**														
**Variable**	IL-17	G-CSF	KC	IL-1β	IL-12/23p40	MCP-1	MIP-1β	IL-4	IL-9	IL-13	RANTES	IL-1α	IL-2	IL-6
**VIP**	1.38	1.26	1.21	1.19	1.17	1.16	1.12	1.10	1.06	1.06	1.05	1.04	1.00	1.00

Although discrimination of colitic from non-colitic mice by non-invasive factors is ideal, as a proof of principle for discriminatory modeling and to gain biological insight into the mucosal disease process *tissue* PLS-DA models were generated using the colon levels of the factors. Compared with the acute *serum* model, the acute *tissue* PLS-DA model was highly discriminatory with 99.1% of the class distinction and 94.8% of the variance accounted for by the components, **Table 1**. Two dominant clusters were present in the VIPs: chemokines, and neutrophil/Th17-associated factors, **Table 2**. The chemokine cluster (MCP-1, MIP-1α, MIP-1β, RANTES, and KC) agrees with the state of active, rather than resolving, acute colitis at 14 DPI in the Cr^+^ mice. The other cluster, neutrophil/Th17-associated factors, included CT, KC, IL-1β, IL-6, IL-12/23p40, G-CSF and IL-17; in concordance with the influx and importance of both Th17 cells and neutrophils in the resolution of *C. rodentium* infection [13], [17], [20], [28]. In the chronic *tissue* model 98.4% of the class distinction and 89.5% of the variance in the factors were explained by the model, demonstrating that non-colitic Hsp^-^ DKO mice could be distinguished from Hsp^+^ DKO colitic mice, **Table 1**. The chronic *tissue* model VIPs, **Table 2**, also include a chemokine cluster (MCP-1, MIP-1β, RANTES, and KC) as well as a neutrophil/Th17-associated cluster (KC, IL-1β, IL-6, IL-12/23p40, and IL-17). The combined findings from colon tissue during acute and chronic colitis indicate that the presence of activated neutrophils and Th17 cells have a strong predictive value for the presence of both acute and chronic colitis.

### Serum neutrophil- and Th17-associated factors discriminate acute from chronic colitis and predict disease severity

**Figure 5:**
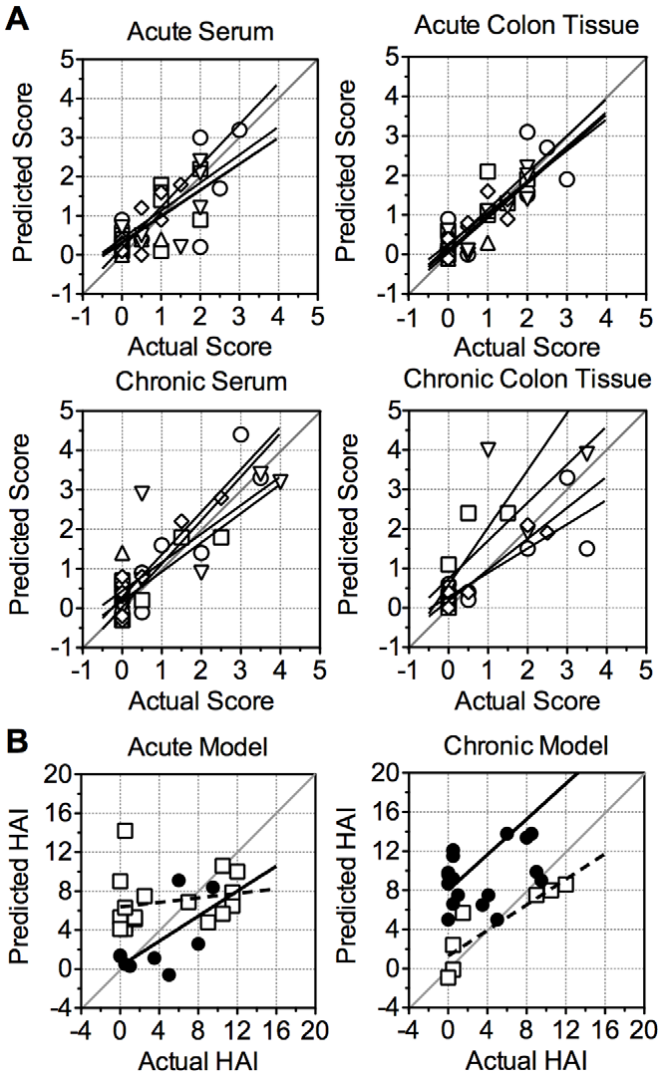
PLS modeling predicts histological lesion scores in colon. (A) Predicted lesion scores from *serum* or *tissue* PLS models using the VIPs of acute or chronic colitis. Lesion scores represented as: inflammation (○), epithelial defects (□), edema (▵), hyperplasia (▿), dysplasia (⋄). (B) Histologic activity indices (HAI, sum of individual lesion scores) for acute or chronic colitis predictions from refined acute or chronic serum PLS models using a minimal set of factors: RANTES, IFN-γ, NT, and G-CSF for acute model or IL-12/23p40, IL-17, G-CSF, CT, and KC for chronic model. Lesion scores or HAI plotted as actual versus predicted with exact predictions lying on the diagonal line (slope of 1). HAI for acute (•) and chronic (□) samples. Lines indicate linear regression of actual versus predicted lesion scores or HAI as representative visualization of Spearman correlations.

Although variables may discriminate between colitic and non-colitic mice, they may not be able to predict the severity of individual lesions (inflammation, epithelial defects, edema, hyperplasia, and dysplasia) in one multivariate model. By generating a PLS model with a training set of data including both X (cytokines/adducts) and Y (lesions) variables, and then using this model on another data set of X variables only (prediction set), the predictive ability of the X variables and the model to output accurate Y values can be assessed. PLS prediction models were generated using the corresponding VIPs for acute colitis *serum* or *tissue* and chronic colitis *serum* or *tissue*. For training and prediction sets, samples were randomly divided into two groups of equal size (n = 8-10) including non-colitic and colitic mice. Despite the small training set size, each of the VIP PLS models were able to accurately predict individual lesion scores and disease severity for their complementary samples, **Figure 5A**. The false positive and false negative rates were low for each of the models: 0/5 and 2/5 in acute *serum,* 0/5 and 0/5 in acute *tissue*, 1/4 and 0/4 in chronic *serum*, and 1/4 and 0/4 in chronic *tissue*. An increased training set size could further increase the accuracy of the models, particularly for the lower range of scores.

Next, to determine whether a smaller set of serum factors were predictive biomarkers of colitis, the serum models were refined to a minimal number of input factors that retained predictive and discriminatory power for intestinal lesion scores. In addition, the models were assessed for their specificity to acute and chronic colitis. Given that colonic disease is generally not isolated to one or two lesions, predicted lesion scores were summed to form predicted HAI. The refined acute *serum* model included: RANTES, NT, G-CSF, and IFN-g. This PLS model had a false positive rate of 0% (0/4) and a false negative rate of 40% (2/5) for acute colitis samples, similar to the larger VIP model, **Figure 5B**. However, when the prediction set was chronic serum samples this acute colitis *serum* model failed to discriminate colitic from non-colitic mice or accurately predict severity, with a false positive rate of 100% (9/9) and a false negative rate of 0% (0/8), **Figure 5B**. Correlation of actual versus predicted HAI confirmed the lack of utility of the acute PLS model with Spearman r's of 0.427 and 0.389 for acute and chronic samples, respectively, and P>0.1 for both sample sets. Therefore, the acute colitis serum biomarker model did not accurately predict either acute or chronic colitis due to a high false negative rate (acute) or false positive rate (chronic).

The predictive chronic colitis *serum* model was refined to consist of G-CSF, KC, IL-17, and IL12/23p40, plus CT as an additional marker specific to neutrophils. This refined model based on chronic serum had a false negative rate of 0% (0 of 3), **Figure 5B**. One mouse with minimal disease was predicted to have moderate colitis, a false positive rate of 25% (1 of 4), however this mouse was Hsp^+^ and would likely develop more severe colitis. Correlation analysis confirmed that the chronic PLS model accurately predicted disease in mice prone to chronic colitis with a Spearman r of 0.991 (P<0.001). To test the specificity of this model for chronic versus acute colitis, acute serum was input into the model. The false positive rate was 100% (4/4) with all uninfected mice predicted to have an HAI>5.0 and the false negative rate was 0% (0/10) as the HAI was over predicted for all mice with acute colitis, **Figure 5B**. Spearman correlation for the predicted HAI of acute samples using the chronic PLS model was not significant (r = 0.417, P = 0.08). This outcome demonstrates the utility of PLS modeling of serum biomarkers in predicting both presence and severity of colitis. In particular, chronic colitis was distinguished from acute colitis via five neutrophil- and Th17-related factors in serum that predict the presence of intestinal disease and severity of lesions even with a small cohort.

## Discussion

Whether caused by microbes or an unknown etiology, the complex cytokine milieu of an intestinal inflammatory response provides a plethora of information about the current disease state. Comprehensive measurement and deconvolution of these potential biomarkers via multivariate analysis allows predictions of disease state and evaluation of therapeutic endpoints. In this proof of principle study immunologic parameters in serum and tissue were evaluated for their utility in modeling and predicting severity of histological lesions and colon disease severity from two forms of microbial-induced colitis: acute *C. rodentium* colitis and chronic *Helicobacter*-dependent colitis.

Tissue-specific cytokine profiling is one approach to diagnosing disease severity, however utility of identified factors as biomarkers of disease is low due to feasibility of repeated sample collection. In mice, tissue cytokine profiling of *C. rodentium*-induced colitis confirmed many previous findings at either the protein or mRNA level. Elevation of other cytokines previously reported in *C. rodentium* colitis, such as IFN-γ and TNF-α, were not detected perhaps due to normalization methods or mRNA versus translated protein abundance [8], [9], [13], [28]. Elevated cytokines in both *C. rodentium*-induced colitis and *Helicobacter*-dependent colitis are also increased in colon tissue of chemically-induced murine colitis, as well as human IBD biopsies [29]–[35]. Acute infectious colitis is often considered a different disease process than chronic spontaneous colitis. This study demonstrates that intestinal inflammation from different etiologies may contain more similarities than differences. The most striking commonality is the presence of neutrophils and Th17 cells, two cell types reciprocally recruited by IL-17, G-CSF, and chemokines [36]-[38]. This reciprocity leads to an unending cycle that can only be disrupted by the clearance of the microbial inducer (i.e. *C. rodentium*, or *Helicobacter spp.*), which does not occur with *Helicobacter spp.* causing chronic inflammation. In both models, the anti-microbial action of neutrophils leads to breakdown of the extracellular matrix and initiation of epithelial repair processes [39], whereas the role of Th17 cells, highly induced in both mucosal infections and autoimmune diseases, is not clear [40], [41]. Maintenance of Th17 cells requires IL-23 that has been shown to be protective against early mortality in *C. rodentium* colitis and necessary for colitis development in *Helicobacter*-dependent colitis [17], [42]. IL-23 appears to protect against early *C. rodentium* mortality via Th17 cell secretion of IL-22 rather than IL-17, which peaks during maximal colitis at 2 WPI [8], [13]. In *Helicobacter*-dependent colitis IL-23 functions to regulate the IFN-γ and IL-17-producing T cell populations that lead ultimately to intestinal inflammation [42]. The precise role of IL-17 is not known in either form of colitis; however its production is associated with other Th17-related cytokines and the presence of neutrophils in the disease process. Similar to murine colitis, neutrophils, Th17 cells, and their related factors (such as IL-17, IL-8, and calprotectin, a product of neutrophil activation) are elevated in IBD patients [36], [43]–[45]. The associative, and likely functional, relationship between neutrophils, Th17 cells, and colitis makes neutrophil- and Th17 cell-related factors reliable biomarkers of murine colitis and candidates as biomarkers for human colitis.

In addition to increased inflammatory cytokines, cell-type specific markers, such as NT and CT, provide information about the presence of macrophages or neutrophils that are commonly found among inflamed tissue in both colitic mice and IBD patients [46]–[49]. As demonstrated in this study stable protein adducts of nitric oxide and hypochlorous acid, NT and CT respectively, are measureable markers of cellular infiltration and activation. Previous studies have demonstrated increased macrophages, iNOS, and serum nitrate/nitrite levels in Cr^+^ mice [8], [9], [22] and *Helicobacter*-dependent colitis [25]. Only serum levels of NT in acute colitis were increased in this study. Interestingly, the colon had constitutively high levels of NT, which may be attributable to continual activation and surveillance of the gut microbial community by resident macrophages and epithelial cells. Unlike NT, CT was present in low levels in both the colon and serum of mice without disease. Tissue levels of chlorotyrosine mirrored many cytokines (i.e. IL-6 and IL-17) that are in low abundance except during active inflammation making it an ideal biomarker of both neutrophil activation and colitis.

Circulating cytokines represent the overall state of the host with contributions from disease sites, lymphoid organs and circulating leukocytes, as well as the liver. Given that blood draining the intestine passes through the liver before further circulation, it is likely that local responses in the liver to intestinal stimuli are a substantial source of circulating cytokines [50], [51]. Serum cytokines may therefore represent the response in the intestines as well as the liver to luminal microbial stimuli and may not be identical to intestinal tissue cytokines. In this study serum cytokine profiling of two colitis models suggest circulating cytokines during acute colitis represent a broader response (perhaps that of both the intestines and liver), whereas serum cytokines during chronic colitis are predominantly those found in the intestinal tissue. Whatever the source of the cytokines, multivariate modeling of serum cytokine profiles predicted both disease presence and severity. One possible caveat is that serum cytokines may not discriminate colitis from other inflammatory diseases with similar systemic responses. Ideally, the serum profiles of multiple diseases would be compared to discriminate between a general inflammatory state and more specific sites of disease such as colitis, bronchitis, or hepatitis.

Many biomarkers in serum or feces have been identified for human IBD with some currently being evaluated for utility in clinical trials [5]. Biomarkers have predominantly been identified individually by association with either CD or UC and include antibodies against self (i.e. perinuclear anti-neutrophil cytoplasmic antibodies [pANCA]), bacterial products (i.e. ASCA, flagellin Cbir1, or *E. coli* OmpC), and glycans [1], [4], [6], [52], [53]. More general, non-antibody biomarkers of inflammation that have been applied to IBD include C-reactive protein and calprotectin [3], [43]. Biomarkers specifically related to the intestinal disease process have not been extensively evaluated. Comprehensive serum profiling of cytokines followed by multivariate modeling, similar to that performed in this study of murine colitis, may identify a panel of disease-associated predictive biomarkers, particularly in individuals identified by genetic screening as having an increased risk for IBD.

Results from this study highlight immunologic similarities amongst two forms of murine colitis, particularly at the disease site. Multivariate modeling demonstrates that a limited set of in vivo measurements from serum or tissue of colitic and non-colitic mice accurately predict the severity of multiple colonic lesions and provide biological insight into factors dominating the disease process. Given that histological evaluation of disease lesions is semi-quantitative and subjective, and the small sample numbers used for predictive modeling, the accuracy of the predictions is encouraging. A larger scale study of the identified biomarkers would further improve the accuracy of the models in predicting severity. Additionally, whether the identified biomarkers are useful as accurate, quantitative markers of complex human diseases is still unknown. Further studies employing multivariate analysis and modeling of in vivo measurements will be useful for discriminating a subset of biologically relevant information from larger data sets, particularly in the setting of translational studies and evaluation of drugs for therapeutic efficacy endpoints.

## Materials and Methods

### Ethics statement

All animal experiments were approved by the IACUC at MIT (0207-020-10) or The University of Chicago (72039).

### Mice and bacteria

For the *C. rodentium* study, female C57BL/6J (6 weeks old) mice were purchased from The Jackson Laboratory (Bar Harbor, ME) and housed at MIT. All TLR4^−/−^ x IL-10^−/−^ (DKO) mice on C57BL/6 background were bred and housed at The University of Chicago. The two DKO colonies originated from an specific pathogen free (SPF) colony at Massachusetts General Hospital (Boston, MA) that was *Helicobacter spp.*-positive (Hsp^+^) by fecal PCR and from which a *Helicobacter spp.*-negative (Hsp^−^) colony was rederived [19]. Male and female 20–35 week-old DKO mice bred and housed in Hsp^−^ or Hsp^+^ SPF facilities at The University of Chicago were utilized. All mice were fed a standard rodent diet and water *ad libitum*, housed in microisolator cages, and maintained SPF, including all known *Helicobacter* spp. (except Hsp^+^ DKO colony), in facilities approved by the Association for Assessment and Accreditation of Laboratory Animal Care. For *C. rodentium* infections, mice were gavaged ∼2×10^9^ kan^r^ *C. rodentium,* and fecal burdens determined as previously described [8]. Presence or absence of *Helicobacter spp.* was confirmed by genus-specific PCR on fecal DNA [54].

### Tissue collection and Histopathology

Serum collected by cardiac puncture at sacrifice was stored at −80°C. For *C. rodentium* studies the colon was removed and divided longitudinally. The distal two-thirds from one section, and the distal quarter from the other were snap frozen in liquid nitrogen and stored at −80°C until protein isolation. Similarly, cecum and colon were collected from the DKO mice and divided longitudinally with one-half snap frozen for protein adduct measurements. A 1 cm piece of proximal colon (1 cm from ileocecal junction) was also collected. All remaining colon or cecum was fixed, sectioned, H&E stained, and scored by a blinded pathologist [8] or stained for MPO enumeration as previously described [25], [55].

### Serum and tissue cytokine measurements

For cytokines colon protein was isolated directly from frozen tissue by homogenization in cell lysis buffer containing phosphotase and protease inhibitors according to manufacturer's protocol (Bio-Rad, Hercules, CA). Cytokines were measured in serum or colon lysates using Bio-Plex Pro Mouse Cytokine 23-plex Assay according to manufacturer's instructions (Bio-Rad, Hercules, CA). Total protein in each colon sample was measured using microBCA assay (Thermo Fischer Scientific Inc., Rockford, IL). Colon cytokines were normalized to total protein concentration of each sample.

### Nitrotyrosine and chlorotyrosine measurements

Nitrotyrosine and chlorotyrosine in serum and colon tissue were measured by negative-ion chemical ionization GC/MS. Briefly, serum or colon protein (2 mg) was spiked with 1 pmol internal standards (L-3-chloro-[^13^C_9_, ^15^N]-tyrosine and L-3-nitro-[^13^C_9_, ^15^N]-tyrosine). Protein was digested by 1 mg Pronase E (Protease from *Streptomyces griseus*, ≥4 units/mg) overnight at pH 7.4, followed by HPLC purification. The purified residue was derivatized with ethyl perfluorobutyrate and N-methyl-N-(t-butyldimethylsilyl) trifluoroacetamide + 1% trimethylchlorosilane (MtBSTFA, Regis Technologies, Morton Grove, IL). The derivatized samples were analyzed by negative-ion chemical ionization GC/MS. Separations were carried out on an Agilent 6890N GCMS system equipped with a 30 m HP-5MS capillary column (0.25 mm I.D., 0.25 µm film thickness). The ions were monitored at *m/z* 489 and 499 for chlorotyrosine and L-3-chloro-[^13^C_9_, ^15^N]-tyrosine, and at *m/z* 518 and 528 for nitrotyrosine and L-3-nitro-[^13^C_9_, ^15^N]-tyrosine. Quantification of protein-bound nitrotyrosine and chlorotyrosine were based on the calibration curves (5-points) constructed over the range of 0.1–5.0 pmol for both nitrotyrosine and chlorotyrosine. All analyses were carried out in triplicate.

### Multivariate Analysis

SIMCA-P+ v11.5 (Umetrics Inc., Kinnelon, New Jersey) software for Partial Least Squares Projection to Latent Structures (PLS)-Discriminant Analysis (DA) was utilized to for the analysis of cytokines, chlorotyrosine and nitrotyrosine levels in colon or serum. Variables were log_10_ transformed as determined necessary by SIMCA-P+ for all analyses. Separate serum and tissue models were generated for each of the murine colitis models. Discrimination was based on assignment of each sample to class 1 (Cr^−^ or Hsp^−^) or class 2 (Cr^+^ or Hsp^+^) for PLS-DA models. R^2^ Y, the fraction of the sum of squares of all Y variables explained by the component of the model, R^2^ Y cumulative, the cumulative sum of squares of all Y variables explained by all components of the model, Q^2^ the fraction of the total variation in Y variables that can be predicted by the component, and Q^2^ cumulative, the cumulative Q^2^ of the Y variables for all components in the model, were used to evaluate the quality of the model. R^2^ cumulative and Q^2^ cumulative of 1 indicate perfect fit and 100% explanation of relationship between X variables and Y variables. Variable importance in the projection (VIP) is computed from influence (weight) on Y of every term in the model. The average VIP equals 1, therefore VIPs >1 explain Y more than VIP <1. For PLS models each group was randomly divided into two sets (training or prediction). Given the small sample size, four different training/prediction sets and models were generated to ensure individual samples were not biasing the models.

### Statistics

Statistical significances were determined by two-way ANOVA followed by Bonferroni post-tests or unpaired two-tailed Students' t test as appropriate. Histologic activity indices were analyzed by Mann-Whitney t test. Acute and chronic model fits of predicted to actual data were analyzed by Spearman nonparametric correlations. GraphPad Prizm Software version 5.0 (La Jolla, CA) was used for all analyses.

## Supporting Information

Figure S1 Peak infection with *C. rodentium* precedes onset of weight loss and disease development. (a) Fecal burden of *C. rodentium* in uninfected (closed square) and infected (open square) mice from Day 0 to Day 14. (b) Percent change in body weight normalized to day 0 to day 14 in uninfected (closed square) and *C. rodentium*-infected (open square) mice. Data are presented as mean ± SEM. *** P<0.001 by two-way ANOVA with Bonferroni post-tests.
(1.25 MB TIF)

Figure S2 Cytokine measurements in C57BL/6J mice with acute infectious colitis. Colon tissue (a) and serum (b) cytokine concentrations in uninfected (Cr^-^; n  =  9 serum, n  =  10 tissue) and *C. rodentium* infected (Cr^+^; n  =  10) mice at 14 DPI. Colon concentrations were normalized to total protein in sample. Bar equals mean value. * P < 0.05, ** P < 0.01, *** P < 0.001 by unpaired Student's T test.
(9.5 MB TIF)

Figure S3 Cytokine measurements in TLR4^-/-^ x IL10^-/-^ (DKO) mice with chronic spontaneous *Helicobacter*-dependent colitis. Colon tissue (a) and serum (b) cytokine concentrations in *Helicobacter* spp.-negative (Hsp^-^; n  =  6) and *Helicobacter* spp.-positive (Hsp^+^; n  =  8 tissue, n  =  10 serum) DKO mice. Colon concentrations were normalized to total protein in sample. Bar equals mean value. * P < 0.05, ** P < 0.01, *** P < 0.001 by unpaired Student's T test.
(9.5 MB TIF)

## References

[1] Sendid B, Quinton JF, Charrier G, Goulet O, Cortot A (1998). Anti-Saccharomyces cerevisiae mannan antibodies in familial Crohn's disease.. Am J Gastroenterol.

[2] Hanauer SB (2010). The expanding role of biologic therapy for IBD.. Nat Rev Gastroenterol Hepatol.

[3] Langhorst J, Elsenbruch S, Koelzer J, Rueffer A, Michalsen A (2008). Noninvasive markers in the assessment of intestinal inflammation in inflammatory bowel diseases: performance of fecal lactoferrin, calprotectin, and PMN-elastase, CRP, and clinical indices.. Am J Gastroenterol.

[4] Dotan I, Fishman S, Dgani Y, Schwartz M, Karban A (2006). Antibodies against laminaribioside and chitobioside are novel serologic markers in Crohn's disease.. Gastroenterology.

[5] Li X, Conklin L, Alex P (2008). New serological biomarkers of inflammatory bowel disease.. World J Gastroenterol.

[6] Lodes MJ, Cong Y, Elson CO, Mohamath R, Landers CJ (2004). Bacterial flagellin is a dominant antigen in Crohn disease.. J Clin Invest.

[7] Borenshtein D, McBee ME, Schauer DB (2008). Utility of the Citrobacter rodentium infection model in laboratory mice.. Curr Opin Gastroenterol.

[8] McBee ME, Zheng PZ, Rogers AB, Fox JG, Schauer DB (2008). Modulation of acute diarrheal illness by persistent bacterial infection.. Infect Immun.

[9] Simmons CP, Goncalves NS, Ghaem-Maghami M, Bajaj-Elliott M, Clare S (2002). Impaired resistance and enhanced pathology during infection with a noninvasive, attaching-effacing enteric bacterial pathogen, Citrobacter rodentium, in mice lacking IL-12 or IFN-gamma.. J Immunol.

[10] Yoshida M, Kobayashi K, Kuo TT, Bry L, Glickman JN (2006). Neonatal Fc receptor for IgG regulates mucosal immune responses to luminal bacteria.. J Clin Invest.

[11] Masuda A, Yoshida M, Shiomi H, Ikezawa S, Takagawa T (2008). Fcgamma receptor regulation of Citrobacter rodentium infection.. Infect Immun.

[12] Simmons CP, Clare S, Ghaem-Maghami M, Uren TK, Rankin J (2003). Central role for B lymphocytes and CD4+ T cells in immunity to infection by the attaching and effacing pathogen Citrobacter rodentium.. Infect Immun.

[13] Zheng Y, Valdez PA, Danilenko DM, Hu Y, Sa SM (2008). Interleukin-22 mediates early host defense against attaching and effacing bacterial pathogens.. Nat Med.

[14] Dann SM, Spehlmann ME, Hammond DC, Iimura M, Hase K (2008). IL-6-dependent mucosal protection prevents establishment of a microbial niche for attaching/effacing lesion-forming enteric bacterial pathogens.. J Immunol.

[15] Gibson DL, Ma C, Rosenberger CM, Bergstrom KS, Valdez Y (2008). Toll-like receptor 2 plays a critical role in maintaining mucosal integrity during Citrobacter rodentium-induced colitis.. Cell Microbiol.

[16] Lebeis SL, Powell KR, Merlin D, Sherman MA, Kalman D (2009). Interleukin-1 receptor signaling protects mice from lethal intestinal damage caused by the attaching and effacing pathogen Citrobacter rodentium.. Infect Immun.

[17] Mangan PR, Harrington LE, O'Quinn DB, Helms WS, Bullard DC (2006). Transforming growth factor-beta induces development of the T(H)17 lineage.. Nature.

[18] Kullberg MC, Ward JM, Gorelick PL, Caspar P, Hieny S (1998). Helicobacter hepaticus triggers colitis in specific-pathogen-free interleukin-10 (IL-10)-deficient mice through an IL-12- and gamma interferon-dependent mechanism.. Infect Immun.

[19] Matharu KS, Mizoguchi E, Cotoner CA, Nguyen DD, Mingle B (2009). Toll-like receptor 4-mediated regulation of spontaneous Helicobacter-dependent colitis in IL-10-deficient mice.. Gastroenterology.

[20] Spehlmann ME, Dann SM, Hruz P, Hanson E, McCole DF (2009). CXCR2-dependent mucosal neutrophil influx protects against colitis-associated diarrhea caused by an attaching/effacing lesion-forming bacterial pathogen.. J Immunol.

[21] Beckman JS, Koppenol WH (1996). Nitric oxide, superoxide, and peroxynitrite: the good, the bad, and ugly.. Am J Physiol.

[22] Vallance BA, Deng W, De Grado M, Chan C, Jacobson K (2002). Modulation of inducible nitric oxide synthase expression by the attaching and effacing bacterial pathogen citrobacter rodentium in infected mice.. Infect Immun.

[23] Gobert AP, Cheng Y, Akhtar M, Mersey BD, Blumberg DR (2004). Protective role of arginase in a mouse model of colitis.. J Immunol.

[24] Chin MP, Schauer DB, Deen WM (2008). Prediction of nitric oxide concentrations in colonic crypts during inflammation.. Nitric Oxide.

[25] Erdman SE, Rao VP, Poutahidis T, Rogers AB, Taylor CL (2009). Nitric oxide and TNF-alpha trigger colonic inflammation and carcinogenesis in Helicobacter hepaticus-infected, Rag2-deficient mice.. Proc Natl Acad Sci U S A.

[26] Harrison JE, Schultz J (1976). Studies on the chlorinating activity of myeloperoxidase.. J Biol Chem.

[27] Domigan NM, Charlton TS, Duncan MW, Winterbourn CC, Kettle AJ (1995). Chlorination of tyrosyl residues in peptides by myeloperoxidase and human neutrophils.. J Biol Chem.

[28] Lebeis SL, Bommarius B, Parkos CA, Sherman MA, Kalman D (2007). TLR signaling mediated by MyD88 is required for a protective innate immune response by neutrophils to Citrobacter rodentium.. J Immunol.

[29] Melgar S, Karlsson A, Michaelsson E (2005). Acute colitis induced by dextran sulfate sodium progresses to chronicity in C57BL/6 but not in BALB/c mice: correlation between symptoms and inflammation.. Am J Physiol Gastrointest Liver Physiol.

[30] Ten Hove T, Corbaz A, Amitai H, Aloni S, Belzer I (2001). Blockade of endogenous IL-18 ameliorates TNBS-induced colitis by decreasing local TNF-alpha production in mice.. Gastroenterology.

[31] Fina D, Sarra M, Fantini MC, Rizzo A, Caruso R (2008). Regulation of gut inflammation and th17 cell response by interleukin-21.. Gastroenterology.

[32] Eastaff-Leung N, Mabarrack N, Barbour A, Cummins A, Barry S (2009). Foxp3(+) Regulatory T Cells, Th17 Effector Cells, and Cytokine Environment in Inflammatory Bowel Disease.. J Clin Immunol.

[33] Banks C, Bateman A, Payne R, Johnson P, Sheron N (2003). Chemokine expression in IBD. Mucosal chemokine expression is unselectively increased in both ulcerative colitis and Crohn's disease.. J Pathol.

[34] Leon AJ, Gomez E, Garrote JA, Bernardo D, Barrera A (2009). High levels of proinflammatory cytokines, but not markers of tissue injury, in unaffected intestinal areas from patients with IBD.. Mediators Inflamm.

[35] Reimund JM, Wittersheim C, Dumont S, Muller CD, Baumann R (1996). Mucosal inflammatory cytokine production by intestinal biopsies in patients with ulcerative colitis and Crohn's disease.. J Clin Immunol.

[36] Pelletier M, Maggi L, Micheletti A, Lazzeri E, Tamassia N (2009). Evidence for a cross-talk between human neutrophils and Th17 cells.. Blood.

[37] Ye P, Rodriguez FH, Kanaly S, Stocking KL, Schurr J (2001). Requirement of interleukin 17 receptor signaling for lung CXC chemokine and granulocyte colony-stimulating factor expression, neutrophil recruitment, and host defense.. J Exp Med.

[38] Wu Q, Martin RJ, Rino JG, Breed R, Torres RM (2007). IL-23-dependent IL-17 production is essential in neutrophil recruitment and activity in mouse lung defense against respiratory Mycoplasma pneumoniae infection.. Microbes Infect.

[39] Nathan C (2006). Neutrophils and immunity: challenges and opportunities.. Nat Rev Immunol.

[40] Steinman L (2009). Mixed results with modulation of TH-17 cells in human autoimmune diseases.. Nat Immunol.

[41] Khader SA, Gaffen SL, Kolls JK (2009). Th17 cells at the crossroads of innate and adaptive immunity against infectious diseases at the mucosa.. Mucosal Immunol.

[42] Kullberg MC, Jankovic D, Feng CG, Hue S, Gorelick PL (2006). IL-23 plays a key role in Helicobacter hepaticus-induced T cell-dependent colitis.. J Exp Med.

[43] Costa F, Mumolo MG, Ceccarelli L, Bellini M, Romano MR (2005). Calprotectin is a stronger predictive marker of relapse in ulcerative colitis than in Crohn's disease.. Gut.

[44] Carlson M, Raab Y, Seveus L, Xu S, Hallgren R (2002). Human neutrophil lipocalin is a unique marker of neutrophil inflammation in ulcerative colitis and proctitis.. Gut.

[45] Fujino S, Andoh A, Bamba S, Ogawa A, Hata K (2003). Increased expression of interleukin 17 in inflammatory bowel disease.. Gut.

[46] Grimm MC, Pavli P, Van de Pol E, Doe WF (1995). Evidence for a CD14+ population of monocytes in inflammatory bowel disease mucosa—implications for pathogenesis.. Clin Exp Immunol.

[47] Grimm MC, Elsbury SK, Pavli P, Doe WF (1996). Interleukin 8: cells of origin in inflammatory bowel disease.. Gut.

[48] Lampinen M, Sangfelt P, Taha Y, Carlson M (2008). Accumulation, activation, and survival of neutrophils in ulcerative colitis: regulation by locally produced factors in the colon and impact of steroid treatment.. Int J Colorectal Dis.

[49] Mitsuyama K, Toyonaga A, Sasaki E, Watanabe K, Tateishi H (1994). IL-8 as an important chemoattractant for neutrophils in ulcerative colitis and Crohn's disease.. Clin Exp Immunol.

[50] Chaluvadi MR, Kinloch RD, Nyagode BA, Richardson TA, Raynor MJ (2009). Regulation of hepatic cytochrome P450 expression in mice with intestinal or systemic infections of citrobacter rodentium.. Drug Metab Dispos.

[51] Tu Z, Bozorgzadeh A, Crispe IN, Orloff MS (2007). The activation state of human intrahepatic lymphocytes.. Clin Exp Immunol.

[52] Satsangi J, Landers CJ, Welsh KI, Koss K, Targan S (1998). The presence of anti-neutrophil antibodies reflects clinical and genetic heterogeneity within inflammatory bowel disease.. Inflamm Bowel Dis.

[53] Joossens S, Colombel JF, Landers C, Poulain D, Geboes K (2006). Anti-outer membrane of porin C and anti-I2 antibodies in indeterminate colitis.. Gut.

[54] Nagamine CM, Rogers AB, Fox JG, Schauer DB (2008). Helicobacter hepaticus promotes azoxymethane-initiated colon tumorigenesis in BALB/c-IL10-deficient mice.. Int J Cancer.

[55] Rogers AB, Cormier KS, Fox JG (2006). Thiol-reactive compounds prevent nonspecific antibody binding in immunohistochemistry.. Lab Invest.

