# Supplementary figures and images for: Multivariate Modeling Identifies Neutrophil- and Th17-Related Factors as Differential Serum Biomarkers of Chronic Murine Colitis
Source: PLoS One. 2010 Oct 19;5(10):e13277. doi: 10.1371/journal.pone.0013277 (PMC2957404; doi:10.1371/journal.pone.0013277)

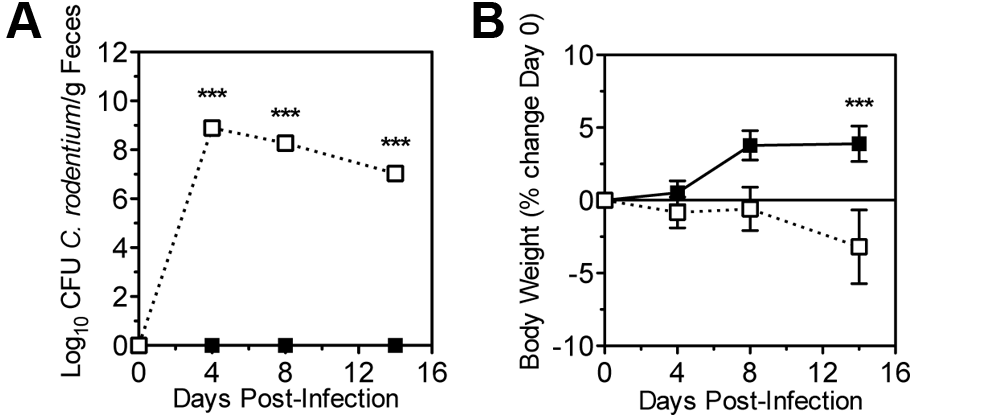

Supplement: Figure S1 — Peak infection with C. rodentium precedes onset of weight loss and disease development. (a) Fecal burden of C. rodentium in uninfected (closed square) and infected (open square) mice from Day 0 to Day 14. (b) Percent change in body weight normalized to day 0 to day 14 in uninfected (closed square) and C. rodentium-infected (open square) mice. Data are presented as mean ± SEM. *** P<0.001 by two-way ANOVA with Bonferroni post-tests. (1.25 MB TIF) [file pone.0013277.s001.tif]

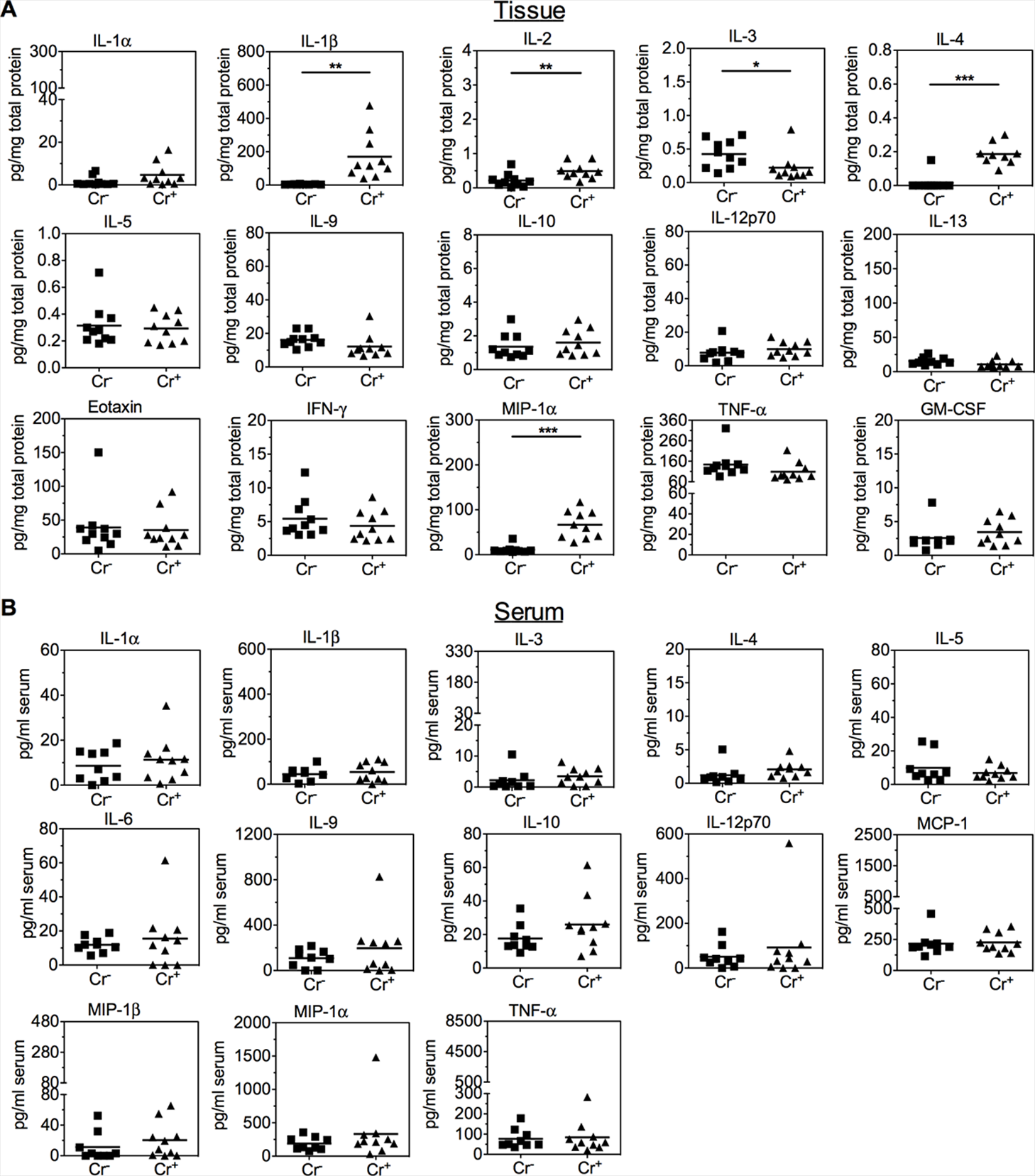

Supplement: Figure S2 — Cytokine measurements in C57BL/6J mice with acute infectious colitis. Colon tissue (a) and serum (b) cytokine concentrations in uninfected (Cr-; n = 9 serum, n = 10 tissue) and C. rodentium infected (Cr+; n = 10) mice at 14 DPI. Colon concentrations were normalized to total protein in sample. Bar equals mean value. * P < 0.05, ** P < 0.01, *** P < 0.001 by unpaired Student's T test. (9.5 MB TIF) [file pone.0013277.s002.tif]

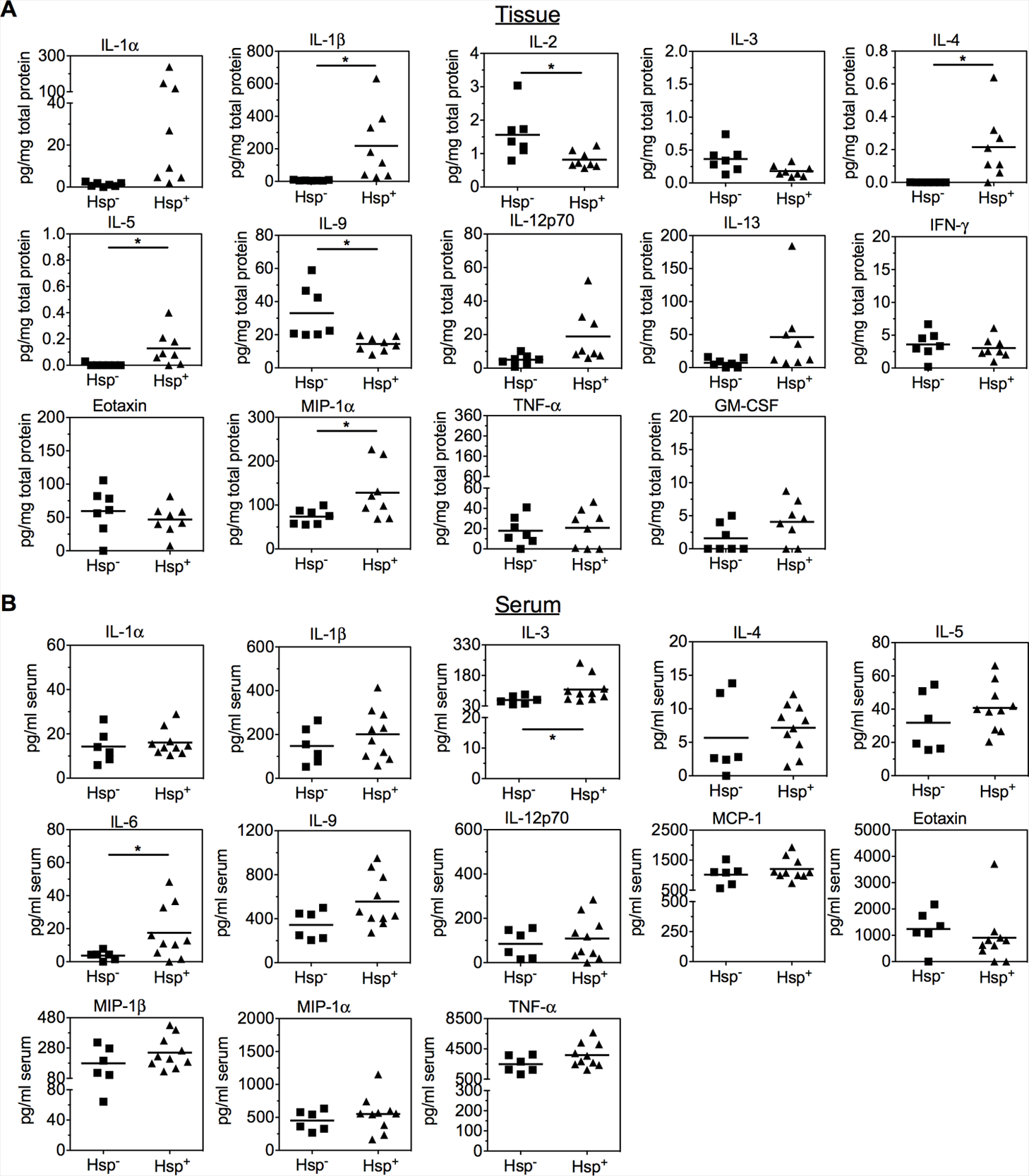

Supplement: Figure S3 — Cytokine measurements in TLR4-/- x IL10-/- (DKO) mice with chronic spontaneous Helicobacter-dependent colitis. Colon tissue (a) and serum (b) cytokine concentrations in Helicobacter spp.-negative (Hsp-; n = 6) and Helicobacter spp.-positive (Hsp+; n = 8 tissue, n = 10 serum) DKO mice. Colon concentrations were normalized to total protein in sample. Bar equals mean value. * P < 0.05, ** P < 0.01, *** P < 0.001 by unpaired Student's T test. (9.5 MB TIF) [file pone.0013277.s003.tif]
